# CT- and MRI-Based 3D Reconstruction of Knee Joint to Assess Cartilage and Bone

**DOI:** 10.3390/diagnostics12020279

**Published:** 2022-01-22

**Authors:** Federica Kiyomi Ciliberti, Lorena Guerrini, Arnar Evgeni Gunnarsson, Marco Recenti, Deborah Jacob, Vincenzo Cangiano, Yonatan Afework Tesfahunegn, Anna Sigríður Islind, Francesco Tortorella, Mariella Tsirilaki, Halldór Jónsson, Paolo Gargiulo, Romain Aubonnet

**Affiliations:** 1 Institute of Biomedical and Neural Engineering, Reykjavik University, 101 Reykjavik, Iceland; f.ciliberti1@studenti.unisa.it (F.K.C.); lorena.guerrini@studio.unibo.it (L.G.); arnareg@ru.is (A.E.G.); marco18@ru.is (M.R.); deborah20@ru.is (D.J.); vincenzocangiano74@gmail.com (V.C.); romaina@ru.is (R.A.); 2 Department of Electrical, Information Engineering and Applied Mathematics, University of Salerno, 84084 Salerno, Italy; ftortorella@unisa.it; 3 Laboratory of Cellular and Molecular Engineering “Silvio Cavalcanti”, Department of Electrical, Electronic and Information Engineering “Guglielmo Marconi” (DEI), University of Bologna, 47521 Cesena, Italy; 4 Department of Engineering, Reykjavik University, 101 Reykjavik, Iceland; yonatant@ru.is; 5 Department of Computer Science, Reykjavik University, 101 Reykjavik, Iceland; annasi@ru.is; 6 Department of Radiology, Landspitali, University Hospital of Iceland, 101 Reykjavik, Iceland; mariella@landspitali.is; 7 Department of Orthopaedics, Landspitali, University Hospital of Iceland, 101 Reykjavik, Iceland; halldor@landspitali.is; 8 Medical Faculty, University of Iceland, 101 Reykjavik, Iceland; 9 Department of Science, Landspitali, University Hospital of Iceland, 101 Reykjavik, Iceland

**Keywords:** knee joint, medical imaging, image segmentation, machine learning, 3D modeling

## Abstract

For the observation of human joint cartilage, X-ray, computed tomography (CT) or magnetic resonance imaging (MRI) are the main diagnostic tools to evaluate pathologies or traumas. The current work introduces a set of novel measurements and 3D features based on MRI and CT data of the knee joint, used to reconstruct bone and cartilages and to assess cartilage condition from a new perspective. Forty-seven subjects presenting a degenerative disease, a traumatic injury or no symptoms or trauma were recruited in this study and scanned using CT and MRI. Using medical imaging software, the bone and cartilage of the knee joint were segmented and 3D reconstructed. Several features such as cartilage density, volume and surface were extracted. Moreover, an investigation was carried out on the distribution of cartilage thickness and curvature analysis to identify new markers of cartilage condition. All the extracted features were used with advanced statistics tools and machine learning to test the ability of our model to predict cartilage conditions. This work is a first step towards the development of a new gold standard of cartilage assessment based on 3D measurements.

## 1. Introduction

Osteoarthritis (OA) is among the most common forms of arthritis [1], occurring when protective hyaline cartilage between bones breaks down through injury or disease. Hyaline cartilage in the knee is an important tissue which, due to avascularity, does not heal spontaneously after injury and often requires surgical intervention. OA of the knee is a major cause of disability worldwide [2], causing significant burden on healthcare systems [3]. The lifetime risk of developing symptomatic knee OA is approximately 45%, with a higher risk being associated with obesity (60.5%) and advancing age [4]. The incidence of OA is predicted to increase in the decades to come due to older populations and obesity [5,6].

OA is a polysymptomatic disease but is generally characterized by thinning and loss of cartilage. Assessment of cartilage condition and thickness is therefore crucial for both detection and monitoring the progression of OA. Diagnosis is usually based on a clinical assessment and a radiographic examination. Planar X-rays of the knee are routinely used in radiographic evaluation, however, soft tissue is not adequately visualized, nor is this modality sensitive to changes in the joint over time [7]. Magnetic resonance imaging (MRI) is the state-of-the-art imaging modality for the assessment of hyaline cartilage and has seen rapid developments in hardware, sequences and image analysis in the last decade [7,8,9,10]. MRI provides a visual assessment of the cartilage and presents a means for quantitative evaluation of the volume and dimensions of the cartilage, and its chemical composition. Comprehensive overviews of MRI sequences for assessing morphological and compositional aspects of knee cartilage are described in [7,8,11]. MRI is capable of accurately assessing the size and thickness of articular cartilage [9,12,13]. In addition to visualizing the cartilage, MRI also images other tissues involved in OA, such as subchondral bone, meniscus and soft tissue. Computed tomography (CT) imaging also provides an excellent 3D representation of cortical bone [14,15,16], osteophytes and soft tissue calcification and has been used to investigate changes in the joint, including trabecular bone remodeling, subchondral cysts and bone sclerosis, all of which can be OA-related changes in the joint [17]. As the understanding of OA develops due to advances in medicine and imaging, it is important that OA is viewed as a disease of the whole organ, involving multiple joint tissues [7].

The severity of OA can be assessed by the degree of joint space narrowing and damage to cartilage and underlying bone. Several scales that exist assess the extent of OA. Kellgren–Lawrence (KL) grading is used for the rating of OA on planar X-rays, where the definite presence of an osteophyte (Kellgren–Lawrence grade 2) confirms a structural diagnosis of OA [18]. Kellgren–Lawrence combines an overall grade for OA from joint space narrowing and the presence of osteophytes, which incorrectly assumes that these structural changes appear continuously [7]. Other grading systems such as the OA Research Society International (OARSI) Atlas system separate a joint space narrowing grade from the presence of osteophytes. Both, however, only assess the tibiofemoral joint, underestimating the patellofemoral contribution to the disease [7]. Other commonly used scales include that developed by Ahlbäck in 1968 [19] which is based on the measurement of joint space narrowing. A 2003 study measuring the inter- and intraobserver reliability of the Ahlbäck scale reported low to medium agreement coefficients, especially when reporting on radiographs of earlier stage OA [20]. Comparisons of knee OA scales have shown moderate correlation with arthroscopic findings and also have moderate to high reliability between individual observers [21]. In a study on severe OA, five radiological grading systems demonstrated medium correlation with intraoperative findings of full-thickness cartilage loss, and moderate interobserver reliability for all systems [22]. In both studies, Kellgren–Lawrence and Ahlbäck showed the highest correlation with cartilage loss, although still in the moderate range. Semi-quantitative MRI-based grading systems such as the Whole Organ Magnetic Resonance Imaging Score (WORMS) and Knee OA Scoring System (KOSS) are based on a variety of features of the MR image from the whole knee joint, including cartilage size and depth, bone marrow lesions and subchondral cysts to name but a few. Some of these semi-quantitative scoring systems have demonstrated ‘within grade’ changes over time, thus exhibiting increased sensitivity compared to traditional grading systems [7]. Quantitative measures using MRI include the cartilage volume and thickness calculated as a continuous variable, requiring segmentation of the cartilage in the MR image. This type of analysis requires high-resolution 3D imaging to facilitate accurate measurements and delineation of defined subregions of the cartilage [7]. Comparison of several clinical, radiographic and biochemical measures revealed that the relatively strongest predictor of longitudinal MRI-defined cartilage thinning was reduced baseline cartilage thickness in the medial femur [23]. Three-dimensional cartilage surface mapping (3D-CaSM) has also shown promise for tracking changes in cartilage thickness, where 6-month changes were observable using the semi-automatic 3D-CaSM algorithm [24]. Recent work in healthy knees has also shown the utility of 3D ultrasound in quantifying cartilage volume when registered with MRI scans [25].

Injury to the knee joint presents a substantial risk of development of OA. Planar X-ray imaging, CT arthrography and MRI have all been used in the assessment of knee trauma following injury [26]. An MRI-based score incorporating traumatic and subsequent degenerative changes was introduced in 2014 by [27]. The Anterior Cruciate Ligament OsteoArthritis Score (ACLOAS) evaluates structural joint damage, features of OA (including cartilage loss) and acute signs of inflammation in traumatic injury to the knee. The ACLOAS aims to be used for longitudinal assessment of injury and subsequent OA in the knee joint.

### 1.1. New Cartilage Assessment Methods/Gold Standard

There are two main volumetric analyses used in the cartilage assessment of this research. The first is a wall thickness analysis, where the cartilage mesh is taken and the thickness of each element is calculated from surface to surface. The hypothesis for this analysis is that patients’ degenerative and traumatic cartilages will be thinner in specific places based on the patient category than the control group. The second is the curvature analysis, which measures the Gaussian curvature of an element based on its surrounding elements. The hypothesis here is that around areas of higher cartilage degradation there will be higher curvature as holes and depressions form around these areas.

### 1.2. Use of 3D Modeling Tools

In this project, the medical 3D modeling software Materialise MIMICS (Materialise Interactive Medical Image Control System, Materialise, Belgium) and 3-Matic were used to analyze the cartilage. The application of the MIMICS software is to segment and isolate the specific cartilages from a CT scan and export the generated 3D mesh for further analysis. MIMICS allows the user to directly extract geometric measurements and densities in Hounsfield unit (HU) values for each element. The exported cartilage parts are subsequently imported into 3-Matic, where components’ meshes are analyzed and features extracted.

### 1.3. Machine Learning and Artificial Intelligence

In the scientific literature, it is possible to find multiple applications of machine learning (ML) and deep learning (DL) starting from MRI or other medical images of the knee related to OA. The progression of OA over time can be predicted with the use of ML algorithms using principal component analysis (PCA) [28], X-ray images and general clinical data [29]. DL was used by Liu et al. [30] to detect acute cartilage injuries within the knee joint, while Bien et al. [31] utilized DL efficiently to detect general abnormalities on knee MRI exams. Moreover, DL was used for OA diagnosis [32] and the prevention of total knee replacement using MRI and non-image features [33]. The KL grade system described above was predicted with DL by Kwon et al. [34], having as initial features the gait data and radiographic images. Moreover, gene expression signatures and ML technologies were used to identify OA through liquid biopsy [35,36]. In this research ML, more specifically tree-based algorithms using novel 3D features proposed here, were implemented for a three-class classification.

This study presents a novel workflow developed in the frame of the EU project RESTORE. It is based on the segmentation and processing of medical images to 3D reconstruct a model of the knee joint. The analysis of these models provides an extensive set of metrics that can be used to assess cartilage and bone condition.

## 2. Materials and Methods

### 2.1. Participants

Participants were recruited as part of the European project RESTORE, (https://restoreproject.eu/, last accessed on 20 January 2022) (CORDIS grant agreement ID: 814558). The aim of RESTORE is to implement patient-specific solutions for cartilage regeneration. This study has been approved by the Icelandic Bioethics Commission (approval number: VSN-19-050).

#### 2.1.1. Recruitment

After completing an informed consent, 47 subjects (24 females, 23 males, mean age = 50 years, std age = 19 years, min age = 20 years, max age = 81 years) underwent X-ray, CT scan and MRI of one knee at Landspitali University Hospital in Reykjavik, Iceland, using standardized acquisition protocols and patient positioning. From the total number of patients, 24 subjects (12 females, 12 males, mean age = 64 years, std age = 12 years, min age = 35 years, max age = 81 years) were suffering from degenerative (D) cartilage. They were on a waiting list for prosthetic replacement. Fifteen subjects (9 females, 6 males, mean age = 35 years, std age = 11 years, min age = 20 years, max age = 50 years) suffered from a knee trauma (T) with possible cartilage injury, and 8 participants (3 females, 5 males, mean age = 34 years, std age = 14 years, min age = 24 years, max age = 67 years) were involved in the study as control (C) subjects (no knee symptoms or history of trauma).

#### 2.1.2. Scanning Process

##### CT Scanner

The CT scanner was a Toshiba Aquillion One, 320 slice, that covered a 16 cm area of interest in a single gantry rotation. Slice thickness was 0.5 mm with an increment of 0.25 mm. Tube voltage was 120 kV, tube current was 250 mA and effective mAs was 125. The protocol covered about 15 cm of area (axial plane) centered at the knee joint with small variations according to patient size. No intravenous contrast was administered. The initial CT dose index was set to 12.1 mGy. The preliminary dose-length product was 193.2 mGy*cm. These values were individually recalculated by the CT scanner for each patient according to size/thickness of the examined area.

##### MRI

The MRI scanner was a 3T Siemens Healthcare Prisma scanner. Volumetric 3D sequences with isotropic voxels of 0.6 mm were acquired in the axial plane with a surface coil without intravenous contrast. This allowed for reconstructions in various planes along regions of interest. A 3D fast spin echo, intermediate weighted and fat-suppressed sequence which allowed for morphologic evaluation of cartilage and for better assessment of subchondral bone marrow was used. The maximum field of view was 16 cm, with a minimum matrix size of 256 × 256. The area of interest was cartilage-covered areas around the knee. The protocol covered 14 cm centered at the knee joint.

### 2.2. Data Processing and Analysis

#### 2.2.1. Segmentation

**Figure 1:**
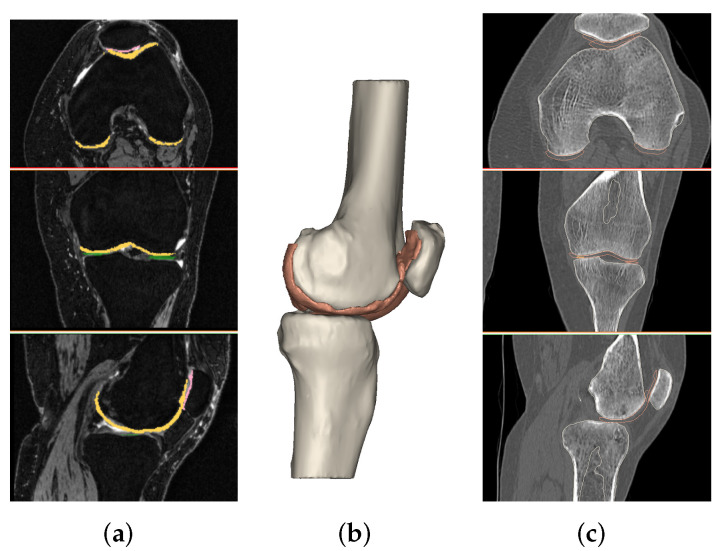
(**a**) Segmentation of femur cartilage from MRI file. (**b**) A 3D model of bones and cartilages. (**c**) Registration on CT file.

All acquired images were processed using medical imaging software, MIMICS, as shown in Figure 1. Knee bones of the femur, tibia and patella and their corresponding cartilages (femoral, lateral tibia, medial tibia and patellar) were considered. The segmentation process was carried out following the same protocols for bones and cartilages, respectively taken from CT scan and MRI.

**Figure 2:**
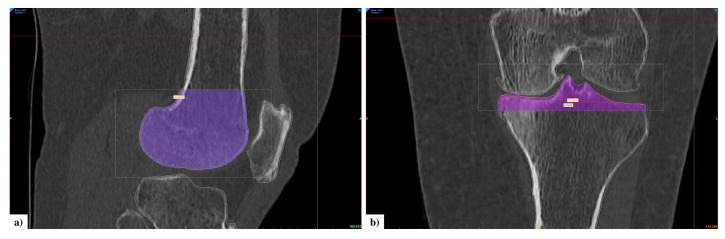
(**a**) Cropped mask of femur bone in sagittal view. (**b**) Cropped mask of tibia bone in coronal view.

The first step was to create a mask for each entity by setting a density threshold interval. If necessary, editing operations were performed to refine the masks and improve their fitting accuracy. These masks were then converted into 3D objects. Next, a new image was created with image registration: MRI and CT images were registered, and bones and cartilage objects were combined. This was carried out by choosing at least four landmarks. The most suitable points are the highest and lowest points on the patella bone in the sagittal view, the upper point of tibial tubercules and the lateral side of the tibia in the coronal view (in any case, other points can also be chosen as landmarks). On the newly created image, a realignment process was manually carried out in order to place bones and cartilages on the correspondent anatomy, aligning the cartilage around the corresponding bone and trying to avoid their overlapping. Once this operation was completed, bone and cartilage masks were created from 3D objects. A manual inspection was carried out to remove any overlap of cartilage over bone. A final visual proof was carried out on 3D models of cartilages. Contact between bones is clearly visible from X-ray images and it shows damage in cartilage tissue. We verified the presence of holes in related objects. Scanned bone segments resulted in different sizes due to the acquisition process. Moreover, our aim was to consider only the part of bone near the cartilages. For these two reasons, we decided to crop femur and tibia bones, selecting a region of interest (Figure 2). The patella is not subject to these issues because it is a small bone, always acquired in its entirety.

MIMICS allows calculation of the radio density in HU directly from a region of interest on CT scans. Human tissues absorb or attenuate X-ray beams according to their density. The HU is a radio density measurement for CT images based on a linear transformation of the X-ray beam attenuation coefficient. Water corresponds to zero on the HU scale, while bones have values higher than 400 [37]. Therefore, cartilage masks were filtered from 0–300 HU, which provides a good range for visualization of soft tissue pixel intensities (cartilage being soft tissue). The cartilage radiodensity was then extracted from the final mask. At the same time, bone mineral density (BMD) was computed from the radiodensity (in HU) using a linear formula that was determined empirically based on a phantom [38]. Volume and surface data were extracted from masks and 3D objects, respectively. Regarding those cartilages that presented holes, we evaluated the extent of the damage using the best fit of an ellipse. We subdivided them into three main categories: grade zero with the absence of holes, grade one when the area is equal to or smaller than 20 mm^2^, grade two when the area is larger than 20 mm^2^.

**Figure 3:**
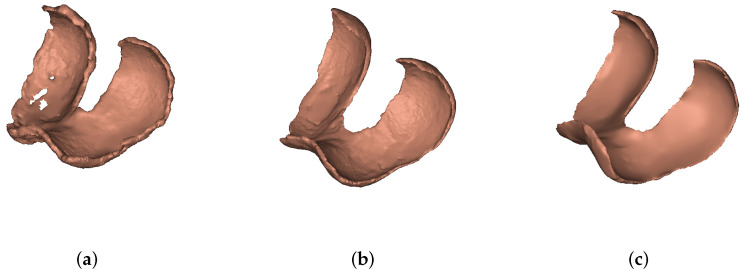
(**a**) Degenerative femoral cartilage, (**b**) traumatic femoral cartilage, (**c**) control femoral cartilage.

Figure 3 shows the 3D computed models of femoral cartilage for each group.

#### 2.2.2. Wall Thickness and Curvature Analysis

After the four cartilages were segmented, a Python script was used to export the parts as Standard Tessellation Language (STL) files to 3-Matic and automatically perform the two analyses for each patient on each cartilage. This resulted in eight analyses per patient which could be further processed for parameter extraction. The analyses gave a result for each element in the STL part file and the parameters extracted were standard deviation (STD), variance (VAR), the mean and root mean square (RMS). Other parameters could also be extracted based on the hypothesis, such as the normalized number of elements below one STD of the mean for the wall thickness analysis and normalized number of elements above one STD of the mean for the curvature analysis. This resulted in 48 parameters per patient. It is important to understand how the results of the analyses were obtained. Each analysis is a text file where each line contains a data point containing the coordinates that make up each triangular element of the STL cartilage file along with a value of thickness or curvature of that element. Since the results were obtained element-wise, one cannot simply take the raw numbers, as the size of each element differs. First, a normalized data set must be calculated, and this can be carried out by applying weights to each result to give it an importance factor. The thickness of a larger element weighs more than the thickness of a smaller one, and the same applies for curvature. As the units for ML methods are somewhat arbitrary, one method for normalization can be to multiply the analysis values by the area of each element. In this manner, it is possible to obtain normalized statistics of the data set. Although the units are different, we still create a standardized form of statistical variance between the patients.

(1) $$Y_{i}=A_{i}X_{i}$$

where $Y_{i}$ is the normalized area result of data point *i* and $X_{i}$ is the ith value of the data point e.g., the curvature of the ith element, and $A_{i}$ is the area of the ith data point.

The next step is to extract statistical features from the normalized data set. Since it is hypothesized that there will be a higher degree of thinner elements in degenerative and traumatic patient cases, it may be interesting to look at the percentage of elements below a certain degree of the mean for the wall thickness analysis following the equation

(2) $$W_{percent}=\frac{\left(N_{E}<\mu_{w}-\alpha \sigma \right)}{N_{E}}$$

where $\mu_{w}$ is obtained for each individual analysis, and is the mean of the standardized data set of the wall thickness analyses. The parameter σ is the standard deviation of each individual analysis. The parameter α is the weight attached to it, and α is also constant for each type of analysis and cartilage, e.g., curvature analyses of the femoral cartilage should all have the same α, and it is obtained through a guess and check method to create the highest possible separation of the patient groups. $N_{E}$ is the total number of elements in each individual analysis. Therefore, $W_{percent}$ is the ratio of the number of elements below a certain degree of the standard deviation of the mean divided by the total number of elements in each individual analysis. A similar equation can represent the curvature analysis, but, instead, it is interesting to look at the percentage of elements above a certain degree of the mean, as a rougher surface should have more curvature,

(3) $$C_{percent}=\frac{\left(N_{E}>\mu_{c}+\alpha \sigma \right)}{N_{E}}$$

where $C_{percent}$ is the ratio of elements, $\mu_{c}$ is the average curvature of the normalized data set and the rest of the parameters are the same as in Equation (2).

### 2.3. Statistical Analysis

A Shapiro–Wilk test was performed to verify the normality of the distribution of the extracted data. Since the tested data were mostly not normally distributed, a Kruskal–Wallis test was conducted on each extracted feature. In cases where the results showed significant differences, a Dunn’s test was used post hoc to analyze the specific sample pairs (C-D, D-T, T-C). The significance level (alpha) was set at 0.05, and the statistical results that present a *p*-value lower than alpha are considered significant. For each comparison, the elements in the sets are the number of patients present in the groups considered (24 in D, 15 in T, 8 in C). Data come from a half-normal distribution, due to the small sample size and the thresholding applied during the segmentation process.

### 2.4. Machine Learning Classification

The aim of the use of ML is to prove the potential ability of these novel 3D features to classify degenerative, traumatic and control subjects. Knime analytics platform was used as it is widely known and gives good performance in biomedical and health engineering applications [39,40,41]. Three tree-based algorithms were selected for the present ML analysis: decision tree (DT), random forest (RF) and gradient boosting (GB) [42,43]. DT simply builds a basic tree while the other two use the DT to improve the power of their predictions. RF combines randomization and bagging while GB applies both bagging and randomization while adding the boosting technique which assigns weights to the classified subjects. Both are known to perform efficiently in multiple health engineering fields [44,45]. K-fold cross-validation was performed during the train and test division with k = 5 folds [46]. Accuracy, specificity and sensitivity for all three classes were used as classification metrics to evaluate the performances of the algorithms.

Different feature selections were given as input to the three tree-based algorithms for a total of 5 with respective numbers in parentheses:

1. Bone (#8): All the features extracted from the knee bones.
2. Cartilage (#16): All the features extracted from the knee cartilages.
3. Bone and Cartilage (B-C) (#24): All the features extracted from the knee bones and cartilages.
4. Wall Thickness and Curvature (WT-C) (#27): All the features explained in Section 2.2.2. Initially, there were 48 of these features, but 13 of them were not considered because their standard deviation was too high compared to the average values, and these data could affect the classification process. The other 8 are the curvature standard deviation weight and the wall standard deviation weight, which are the same for every subject.
5. Total Features (TOT) (#51): B-C and WT-C features together.

Hole-related features have not been considered because most of them are zero, so they do not add any useful information for the classification.

A feature importance calculation was performed after the classification process for the RF algorithm model.

## 3. Results

### 3.1. 3D Measurements

The main damage from injury or disease in the cartilage tissue is highlighted by alterations in the composition of the synovial joint and surface degeneration, remodeling of subchondral bone, formation of osteophytes and appearance of holes in cartilage [47].

The data shown in Table 1 display the average bone mineral density, as well as radiodensity, volume and surface from each cartilage after tissue segmentation. The results were calculated for each group (degenerative, traumatic and control) and compared.

**Figure 4:**
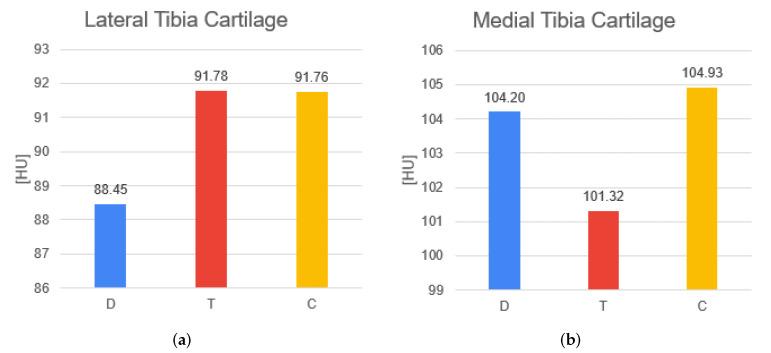
Comparison of the HU values for the two parts of tibia cartilage for D, T, C groups. (**a**) shows the average density value for the lateral tibia cartilage. (**b**) shows the average density value for the medial tibia cartilage. The results are the same as in Table 1.

The D group presents lower BMD values compared to the others. The T and C groups have same values of density for patella bones, while for femur and tibia, the T group has higher values. Significant statistical results are between T–D groups both for the femur (*p*-value = 0.000352) and for tibia (*p*-value = 0.00294); for the patella between C–D groups (*p*-value = 0.0236) and T–D groups (*p*-value = 0.00044). The T group has the lowest volume and the highest surface in the patella. The D and C groups show similar trends in the volume and surface for the patella. However, no significant evidence comes from patella volume and surface. The C group presents higher HU values for all cartilage segments. The T and D groups differ from case to case for every cartilage. The D and T groups have inferior values in the femoral cartilage and patella cartilage. However, the only significant difference in density is in the patella cartilage among C–D groups (*p*-value = 0.0144). Medial and lateral tibial cartilage have opposite HU behaviors in T and D conditions (Figure 4), while these values remain constant in the C group. For all cartilages, volumes are higher in the D group compared to the other groups. The T group has a higher volume than the C group for the femoral and tibia cartilage. In patella cartilage, the volume is higher in the C group. Surface results are also higher in all cartilage parts for the D group. Moreover, the volume and surface of femoral cartilage demonstrate significant differences: significant differences in the volume are present among C–D (*p*-value = 0.00212) and T–D groups (*p*-value = 0.0055) and on the surface only between T–D (*p*-value = 0.0143). For all other features no significant results appear.

Holes are detected in eight patients of the D group with the damage mainly in the femoral cartilage, followed by tibia and patella cartilage, as shown in Table 2 and Table 3. Only one traumatic patient presents several holes.

### 3.2. Wall Thickness and Curvature Analyses

The two analyses were run on each patient’s femoral, patellar, medial and lateral tibial cartilages, resulting in eight data files for each patient. Each row of the data files contains three (x,y,z) coordinates in millimeters defining the element and the analysis value for that element. After the element values were normalized according to Equation (1), the variables described in Section 2.2.2 were extracted.

**Figure 5:**
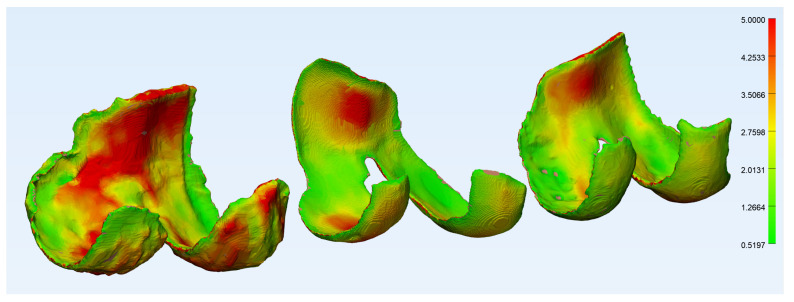
The wall thickness analysis of a femoral cartilage in 3-Matic, degenerative group (**left**), traumatic group (**center**) and control group (**right**).

**Figure 6:**
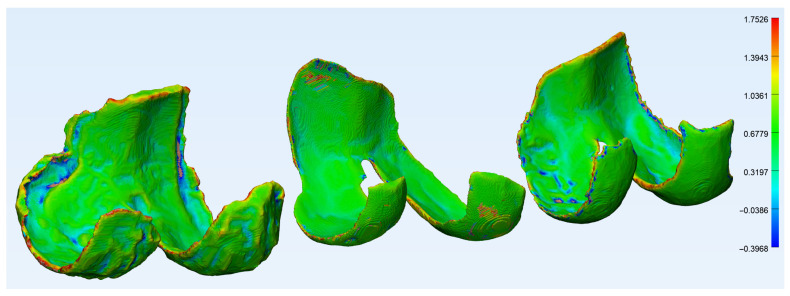
The curvature analysis of a femoral cartilage in 3-Matic, degenerative group (**left**), traumatic group (**center**) and control group (**right**).

Figure 5 and Figure 6 show the wall thickness and curvature analyses of a femoral cartilage, respectively. The wall thickness analysis shows the distribution of thickness and one can see the very thin areas, especially around the holes in the cartilage. The curvature analysis shows the distribution of curvature about the cartilage, and most of the regions of high curvature are around the edges. There are a few elements in the regions around the holes with high curvature, but they are badly represented as the edges around the holes are very thin and therefore contain very few elements.

**Figure 7:**
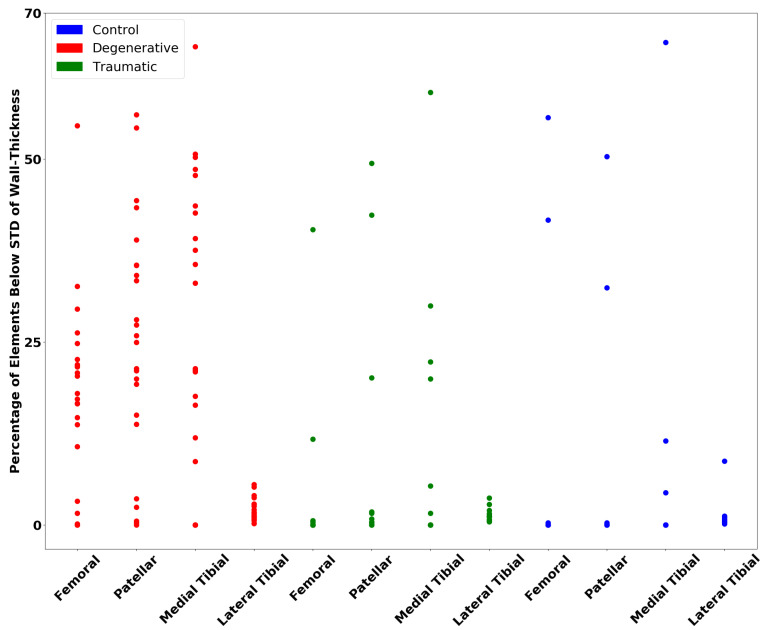
Percentage of elements below a certain standard deviation of the mean, using an STD weight of 0.5 for femoral, patellar and medial tibia cartilages and 0.3 for the lateral tibia cartilage as per Equation (2).

Figure 7 shows the number of elements below 0.5 STD of the mean wall thickness for the femoral, patellar and medial cartilages and 0.3 for the lateral. These weights on the standard deviation gave a consistent separation of the traumatic and degenerative patients from the control patients.

**Figure 8:**
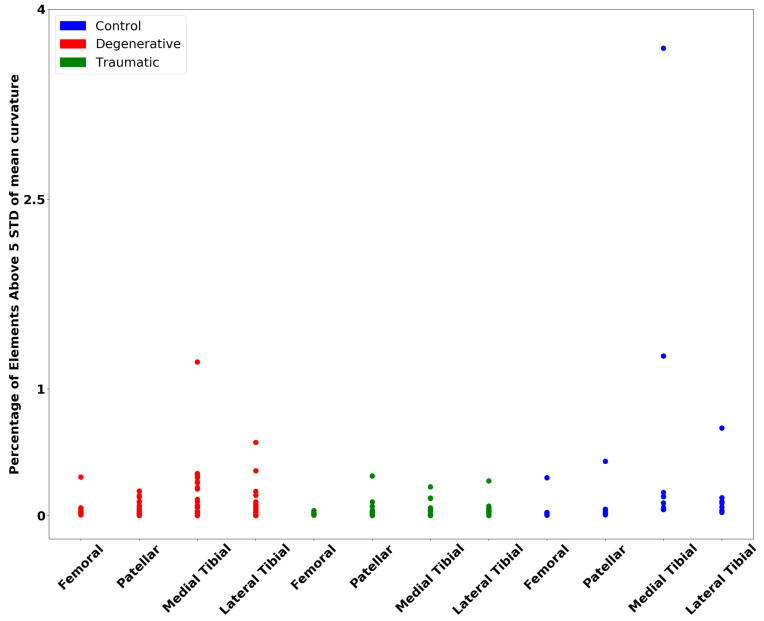
Percentage of elements above a certain standard deviation of the mean, using an STD weight of 5 for all cartilages as per Equation (3).

Figure 8 shows elements above 5 STD of the mean curvature for all cartilages. It was a bit more difficult to find a weight on the STD for the curvature to create a separation of the groups, but one can see that there is some separation, at least for the degenerative patient group.

### 3.3. Machine Learning

Table 4 shows the evaluation metrics for the three-class classification of degenerative, traumatic and control subjects using the five different feature selections described in the previous section. The best accuracy is 76.1 obtained with the B-C and bone selection performing the RF algorithms, which gives the best accuracy in all the models. The TOT selection gives an accuracy higher than 70, while cartilage and WT-C can reach a maximum of 69.6 and 63.0, respectively. The highest sensitivity is always obtained for the degenerative subjects, while the lowest is for the control subjects, having a maximum of 50. Traumatic subject sensitivity is between the other two, having a maximum of 87.5 with the RF algorithm and bone selection. Specificity is generally high, having a value of around 90 for all the control patients.

The feature importance obtained with the RF algorithm can be seen in Table 5: it is clear that the volume of the femoral cartilage (FemCartVOL) has high influence for all the selections in which it is present. Patella density (PatellaDENS) is also important in the multi-class classification with the TOT and B-C feature selection. The WT-C selection was not considered in this analysis because of the low accuracy obtained (maximum of 63.0) (Table 4), but it can be noticed that some of its features have quite good importance in the TOT model (Table 5).

## 4. Discussion

This work aims to develop a new workflow to assess cartilage condition. Based on CT and MRI scans, a complete segmentation of the knee is performed. A 3D model is reconstructed from this segmentation, from which a unique set of data is extracted.

The relationship between systemic bone mineral density (BMD) and cartilage properties has been enhanced in recent years. Previous reviews indicate the positive correlation between BMD and cartilage defects, particularly related to the knee joint [48,49]. The results of this study show the degenerative group presenting lower BMD values compared to healthy and traumatic individuals. Although several studies have demonstrated that higher systemic BMD is associated with increased progression of cartilage defects, this relationship is still under investigation [50]. A recent study demonstrated that BMD was the highest when the knee OA was mild, and was significantly lower in moderate and severe OA. Generally, it is considered that the severity of OA increases and the level of BMD decreases with increasing age [51]. The different correlations between data could be due to the restricted number of participants, their characteristics, outcome measures or the status of the knee joint (as it is in the early stage). However, BMD could be an index of the cartilage condition because it is now validated that bone condition affects the course of the most common joint disease. In summary, the indexes based on the bone could show a way to differentiate the groups. This is proved by the conducted statistical analysis: for the patella bone, the D group can be discernible from C and T groups, and for the femur and tibia bones, it is differentiated from the T group. Moreover, the T group shows lower patella volume than the other groups. Some trauma may have involved the patella and caused its dislocation and subsequent cartilage lesion, leading to early OA [52]. Thus, the examination of the patella volume can be of interest to investigate the presence of traumas.

Still, regarding the patella, its cartilage density is a good discriminator between C and D groups. In general, control patients present higher HU values for all cartilage segments, while those of traumatic and degenerative groups change from case to case. This could be because of the fact that cartilages in the early stages of OA generally present a greater amount of water with respect to physiologically normal cartilages [53]. Therefore, the density calculated for the entire cartilage volume can discriminate between pathological and healthy conditions. Since traumatic and degenerative patients have opposite HU behaviors in lateral and medial tibial cartilages, while control ones remain constant for both parts, this result could represent proof of control regarding tissue density and pathologies.

The measurements of cartilages show a characteristic trend: volume increases for all the cartilages in degenerative patients. This is also strongly confirmed by the significant difference between the degenerative group and the other two groups for the femoral cartilage. Greater volume may indicate cartilage swelling in the early stage of degeneration due to an increased water content [54,55]. This is in agreement with biomechanical evidence, which suggests that the volume of degenerative cartilages and surrounding tissues would be greater than in healthy and traumatic circumstances. Moreover, all degenerative surface cartilage results were higher and we found a corresponding statistical difference between traumatic ones regarding the femoral cartilage. It is particularly interesting how the patella bone and cartilage play a fundamental role in the diagnosis. Until now, the evaluation of cartilage has been conducted mainly on the tibiofemoral joint, and the predominant scales for cartilage diseases do not take into account the patellofemoral compartment [7]. Our study reveals that this patellar joint should be considered with more interest in knee assessment.

The visual inspection of the 3D models reveals that holes are present only in the degenerative cartilage. A third of the patients in the degenerative group present holes in their cartilage, mainly in the femur. Despite the limited amount of data analyzed in this paper, the fact that holes are only present in the degenerative group establishes groundwork for future research with aim of demonstrating that the detection of holes can be an indicator of degenerative cartilage.

The wall thickness analysis presented results of interest. A clear separation of the degenerative and traumatic patients from the control group is visible. Although a more significant separation was expected for the traumatic group, the wall thickness analysis did not take into account the holes that formed in the cartilages, which should be considered, as they are a form of degeneration. This is supported by Vincent and Wann [56], as they have found a link between trauma to cartilage and a decreased wall thickness, as seen much more clearly in the degenerative case. The curvature analysis shows less clear results, although a separation for the degenerative group can be observed. Studies such as that of Folkesson et al. [57] have found that the curvature for patients suffering from OA is significantly higher when compared to control patients, which is shown in the results here, as OA is a degenerative disease. Although it might be expected to find similar trends in the traumatic patients, the analysis results do not show as noteworthy a difference.

Most of the ML results can be considered highly satisfactory: it is clear that the models, especially using RF, can predict with good sensitivity the degenerative and traumatic subjects, and all the 3D novel features extracted appear to have a good predictive power (excluding the WT-C selection, whose results are lower). This can also be seen from the feature importance results (Table 5), where cartilage and bone features assume a dominant role in the classification. To our knowledge, these features extracted manually from bones and cartilage images of the knee have never been used to distinguish the type of injury that provokes OA. A similar accuracy was obtained by Kwon et al. [34] using gait and X-ray data (75.5%) predicting KL grade, while Du et al. [28] using a PCA approach had a similar sensitivity with RF in the KL grade classification, achieving better results with other algorithms with respect to RF, such as simple artificial neural networks or support vector machines. A future improvement of the study can be to extend the classification to existing evaluation grades such as KL, which, knowing the current results, can give us interesting results in terms of accuracy and sensitivity and eventually other algorithms such as simple or advanced artificial neural networks can be used. A new index can also be developed, starting from these results, by integrating the 3D ones and other features extracted from the 2D image manual elaboration of the CT scans and MRI knee exams, for example, bone osteonecrosis, sclerosis, osteophytes and others.

### Limitations

This work presents some limitations. The number of patients is limited. Moreover, the three categories are unbalanced: there are currently a lot of degenerative subjects and few control subjects. This affects the specificity results in the ML classification for the control subjects; in the future, the application of algorithms that can balance the number of subjects of the different classes (i.e., SMOTE [58]) can be considered and, generally speaking, the results would be more accurate with an increased number of samples. More samples will eventually allow the use of more advanced artificial intelligence technologies such as DL and advanced neural networks. In addition, to improve the accuracy, the control patients could be excluded, and a binary classification can be performed on degenerative and traumatic patients.

The segmentation was carried out manually, leading to possible inaccuracies in the elements’ conformation. Although a standard protocol was defined, the overall procedure was performed by different people, and, occasionally, decisions were made based on visual interpretations. Therefore, some inaccuracies may be present in the final data.

Image quality also affects the initial steps of the segmentation process and the subsequent data extraction.

The wall thickness analysis also did not take into account any holes in the cartilage; this leaves out valuable information that could be extracted from the cartilage models as the traumatic patient group often had gaps in their cartilage. This could explain the relatively low percentage of elements below the standard deviation of the mean in Figure 7 compared to the degenerative patients.

## 5. Conclusions

The research focuses on the realization of a new methodology to assess cartilage condition, based on density and geometric features from 3D reconstructed knee joints. Both bone and cartilage metrics revealed interesting indicators to evaluate cartilage status. For the bones, it is shown that bone mineral density is related to cartilage integrity. Additionally, patellar bone volume can help to differentiate healthy knees from traumatic ones. Regarding cartilage, the radiodensity of cartilage can be a robust index to distinguish pathological and healthy conditions. Similarly, cartilage volume can show cartilage degeneration. An in-depth analysis of cartilage geometries showed that thickness and curvature analyses are powerful tools to discriminate healthy patients from patients suffering from a pathological condition. The patellofemoral compartment should be investigated in more depth to evaluate the knee joint condition. Those individual results combined have shown the potential to be reliable indicators of knee condition. Advanced statistics and machine learning demonstrated that it is possible to classify the knee cartilage status based on the previously extracted features. This study is a step towards defining solid indicators of knee joint quality. Pursuing this research by adding new parameters such as age and gender, and also longitudinally observing the evolution of the presented knee joints through time, would be interesting to confirm and identify more accurate markers of cartilage health.

## Figures and Tables

**Table 1:** The 3D results. Mean is reported for each group. Standard deviation is reported in brackets and median in the row below in bold.

	D	T	C
**Bone Mineral Density (g/cm^3^)**			
Femur	1.25 (0.03)	1.29 (0.03)	1.27 (0.03)
	**1.25**	**1.30**	**1.27**
Tibia	1.28 (0.02)	1.32 (0.03)	1.30 (0.03)
	**1.29**	**1.32**	**1.30**
Patella	1.34 (0.05)	1.41 (0.04)	1.41 (0.05)
	**1.35**	**1.42**	**1.40**
**Patella Volume (mm^3^)**	20,368.41 (4486.86)	17,747.93 (4389.93)	19,375.79 (5009.31)
	**19,315.82**	**16,575.77**	**20,508.23**
**Patella Surface (mm^2^)**	4867.88 (1614.10)	5648.82 (3180.33)	4105.83 (725.54)
	**4402.45**	**4516.32**	**4234.59**
**Radiodensity (HU)**			
Femur Cartilage	88.55 (5.74)	88.64 (12.34)	94.06 (7.45)
	**89.51**	**86.56**	**97.17**
Lateral Tibia Cartilage	88.45 (8.30)	91.78 (19.85)	91.76 (3.10)
	**88.20**	**89.53**	**92.21**
Medial Tibia Cartilage	104.20 (19.94)	101.32 (16.70)	104.93 (7.65)
	**98.90**	**97.33**	**103.74**
Patella Cartilage	78.98 (17.53)	79.19 (18.27)	95.10 (16.47)
	**74.94**	**77.16**	**88.36**
**Cartilage Volume (mm^3^)**			
Femur Cartilage	20,265.18 (6856.78)	13,429.59 (2725.04)	11,764.49 (4479.56)
	**19,618.83**	**12,956.89**	**9654.09**
Lateral Tibia Cartilage	2075.11 (1515.56)	1110.60 (409.59)	757.59 (380.87)
	**1371.79**	**1160.67**	**725.18**
Medial Tibia Cartilage	1526.14 (1226.54)	981.93 (610.23)	555.76 (368.51)
	**1067.28**	**868.62**	**440.17**
Patella Cartilage	3241.89 (1164.37)	2778.97 (656.93)	2866.61 (715.97)
	**3199.50**	**2734.05**	**2854.35**
**Cartilage Surface (mm^2^)**			
Femur Cartilage	14,737.79 (2866.13)	12,270.98 (1378.72)	11,968.50 (2663.58)
	**14,076.96**	**12,319.56**	**11,541.29**
Lateral Tibia Cartilage	1809.96 (922.87)	1299.96 (437.06)	1082.64 (371.60)
	**1458.98**	**1238.18**	**1038.20**
Medial Tibia Cartilage	1702.80 (997.76)	1233.47 (469.07)	949.80 (400.35)
	**1288.59**	**1172.29**	**1001.64**
Patella Cartilage	2546.89 (469.92)	2443.72 (480.52)	2517.28 (409.93)
	**2588.05**	**2371.60**	**2530.24**
**Presence of Holes (%)**	3.76	0.47	0

**Table 2:** Number of holes.

Patients	Femoral Cartilage	Lateral Tibia Cartilage	Medial Tibia Cartilage	Patella Cartilage
1 (D)	3	1	7	0
2 (D)	2	0	0	0
3 (D)	1	0	1	0
4 (D)	1	0	0	1
5 (D)	1	0	0	3
6 (D)	4	0	0	0
7 (D)	1	0	0	0
8 (D)	1	0	0	0
9 (T)	5	0	0	2

**Table 3:** Total hole surface.

Patients	Femoral Cartilage (mm^2^)	Lateral Tibia Cartilage (mm^2^)	Medial Tibia Cartilage (mm^2^)	Patella Cartilage (mm^2^)
1 (D)	50.11	1.18	53.79	0.00
2 (D)	186.83	0.00	0.00	0.00
3 (D)	29.57	0.00	22.91	0.00
4 (D)	3.06	0.00	0.00	2.97
5 (D)	10.52	0.00	0.00	4.03
6 (D)	19.80	0.00	0.00	0.00
7 (D)	0.78	0.00	0.00	0.00
8 (D)	4.60	0.00	0.00	0.00
9 (T)	488.81	0.00	0.00	1.87

**Table 4:** Classification metrics for the three-class classification (degenerative (D), traumatic (T), control (C)) using the five different feature selections.

Feat. Selection	Alg.	Acc.	Sens D	Spec D	Sens T	Spec T	Sens C	Spec C
TOT	RF	71.7	87.5	63.6	57.1	93.8	50.0	92.1
	GB	67.4	87.5	68.2	64.3	84.4	12.5	92.1
	DT	63.0	79.2	59.1	71.4	78.1	0.00	97.4
B-C	RF	**76.1**	87.5	68.2	85.7	90.6	25.0	97.4
	GB	69.9	79.2	72.7	71.4	90.6	37.5	86.8
	DT	58.7	66.7	77.3	78.6	65.6	0.00	92.2
Bone	RF	**76.1**	91.7	72.7	87.5	87.5	12.5	91.4
	GB	67.4	83.3	68.2	78.6	78.1	0.00	97.4
	DT	60.9	79.2	63.6	50.0	81.2	25.0	89.5
Cartilage	RF	69.6	87.5	59.1	50.0	90.6	50.0	94.7
	GB	63.0	75.0	77.3	57.1	81.2	37.5	84.2
	DT	63.0	75.0	86.4	57.1	71.9	37.5	86.6
WT-C	RF	63.0	83.3	77.3	57.1	75.0	12.5	89.5
	GB	60.9	75.0	77.3	57.1	75.0	28.6	86.8
	DT	60.9	75.0	72.7	57.1	81.2	25.0	84.2

**Table 5:** Feature Importance: The most important features for all the feature selections (excluding WT-C) for the RF algorithm classification model (12 features for the TOT and B-C selections out of 51 and 24, respectively). The percentage of importance for each parameter in the corresponding feature selection is shown. All eight features are presented for the bone selection and eight out of sixteen for the cartilage selection. “DENS” stands for density and when “Cart” is not mentioned in the name of the feature it means that the bone value is considered. STD refers to the DENS values.

Impo	TOT	%	B-C	%
1	FemCartVOL	6.69	PatellaDENS	8.54
2	PatellaDENS	5.69	FemCartVOL	7.91
3	FemWallBelowSTDWeight	4.22	FemCartSURF	7.28
4	FemCartDENS	3.77	TibiaDENS	6.16
5	PatWallVar	3.65	PatCartDENS	5.91
6	LatWallMean	3.45	TibCartLatVOL	5.41
7	PatCartDENS	3.41	PatellaSTD	5.35
8	FemWallRMS	3.16	PatellaSURF	4.72
9	PatWallBelowSTDweight	3.13	FemCartDENS	4.51
10	TibiaDENS	3.06	TibCartLatSURF	4.37
11	FemCartSTD	3.01	PatellaVOL	4.35
12	FemWallMean	2.82	TibCartMedSURF	4.30
**Impo**	**Bone**	**%**	**Cart**	**%**
1	FemurDENS	17.74	FemCartVOL	14.21
2	FemurSTD	16.87	FemCartDENS	11.07
3	TibiaDENS	15.20	PatCartDENS	9.31
4	TibiaSTD	12.57	FemCartSURF	8.14
5	PatellaDENS	12.21	TibCartLatVOL	6.84
6	PatellaSTD	10.24	TibCartMedSTD	6.51
7	PatellaVOL	7.69	TibCartLatDENS	5.96
8	PatellaSURF	7.48	FemCartSTD	5.85

## Data Availability

In the year 2022, an online database will be available with the data used and processed for this study, through the following website: https://restore-project.ru.is/, accessed on 20 January 2022.

## Abbreviations

The following abbreviations are used in this manuscript:

B-C	Bone and Cartilage
C	Control Group
CT	Computed Tomography
D	Degenerative Group
DL	Deep Learning
DT	Decision Tree
GB	Gradient Boosting
HU	Hounsfield Unit
ML	Machine Learning
MRI	Magnetic Resonance Imaging
OA	Osteoarthritis
OARSI	Osteoarthritis Research Society International
RF	Random Forest
STL	Standard Tessellation Language
T	Traumatic Group
TOT	Total Features
WT-C	Wall Thickness and Curvature
